# Real-Time and Secure Wireless Health Monitoring

**DOI:** 10.1155/2008/135808

**Published:** 2008-05-11

**Authors:** S. Dağtaş, G. Pekhteryev, Z. Şahinoğlu, H. Çam, N. Challa

**Affiliations:** ^1^Department of Information Science, University Arkansas, Little Rock, AR 72204-1099, USA; ^2^Teknoset Research, TUBITAK MAM, Gebze 41730, Turkey; ^3^Mitsubishi Electric Research Labs, 201 Broadway Avenue, Cambridge, MA 02139, USA; ^4^Computer Science and Engineering Department, School of Engineering Arizona State University Tempe, Arizona State University, Tempe, Arizona 85287, USA

## Abstract

We present a framework for a wireless health monitoring system using wireless networks such as ZigBee. Vital signals are collected and processed using a 3-tiered architecture. The first stage is the mobile device carried on the body that runs a number of wired and wireless probes. This device is also designed to perform some basic processing such as the heart rate and fatal failure detection. At the second stage, further processing is performed by a local server using the raw data transmitted by the mobile device continuously. The raw data is also stored at this server. The processed data as well as the analysis results are then transmitted to the service provider center for diagnostic reviews as well as storage. The main advantages of the proposed framework are (1) the ability to detect signals wirelessly within a body sensor network (BSN), (2) low-power and reliable data transmission through ZigBee network nodes, (3) secure transmission of medical data over BSN, (4) efficient channel allocation for medical data transmission over wireless networks, and (5) optimized analysis of data using an adaptive architecture that maximizes the utility of processing and computational capacity at each platform.

## 1. INTRODUCTION

As numerous wireless personal area networking (WPAN) technologies emerge, the interest for applications such as health monitoring, smart homes, and industrial control has grown significantly. ZigBee is the first industrial standard WPAN technology [1] that provides short-range, low-power, and low-data-rate communication, and supports mesh networking and multihopping. While many smart home application areas such as lighting, security, and climate control have been suggested using the ZigBee standard, health-care applications have not received much attention despite their importance and high-value added. Here, we present a wireless communication system for real-time health monitoring with secure transmission capability.

Early clinical trials conducted in the mid-nineties by the National Institute of Health in the Mobile Telemedicine project [2] has indicated that the transmission of critical patient data during emergencies can make significant difference in patient outcomes. This result has led to a proliferation of health-care projects, including CodeBlue [3], PPMIM [4], CustoMed [5], MobiCare [6], LiveNet [7], PCN [8], UbiMon [9], MobiHealth [10], AMON [11], and PadNET [12]. Various types of wearable health monitoring sensor devices have been developed and integrated into patients' clothing [13–16], an armband [17], or wristband [18].

Most of the existing mobile patient monitoring projects such as PPMIM [4], MobiCare [6], MobiHealth [10], and AMON [11] employ cellular networks (e.g., GSM, GPRS, or UMTS) to transmit vital signs from BSN to health centers. For instance, in PPMIM project, a remote medical monitoring three-tier architecture with a GSM/GPRS peer-to-peer channel is presented, and the concept of multiresolution is introduced to identify useful information and to reduce communication costs. In MobiCare, a body sensor network (or MobiCare client) and health-care servers employ short-range Bluetooth between BSN and BSN Manager, and GPRS/UMTS cellular networks between BSN Manager and health-care providers. The UbiMon (Ubiquitous Monitoring Environment for Wearable and Implantable Sensors) Project aims to provide a continuous and unobtrusive monitoring system for patients in order to capture transient but life threatening events [19]. Among major projects in the area, CodeBlue [20] is the only existing project that employs wireless sensor networks in emergency medical care, hospitals, and disaster areas as an emergency message delivery system. With MICA motes [21], CodeBlue uses pulse oximetry and electrocardiogram (ECG) sensors to monitor and record blood oxygen and cardiac information from a large number of patients. However, most of the existing systems lack two key features: (1) wireless communication technology that conforms to standards, (2) integration with wireless sensor network platforms such as smart home systems, and (3) secure transmission capability that addresses the resource constraints optimally.

Our ZigBee-based architecture is based on the premise that the secure wireless communications combined with the widespread infrastructure provided by applications such as smart homes will be key to the effective use of future medical monitoring systems. This is due to the fact that practicality of the sensing, transmission, and processing steps is often the major obstacle against common use of such devices. Therefore, we believe that medical monitoring based on the emerging smart home wireless technology, ZigBee, has a great potential.

In addition, optimized processing of the collected data plays a key role. With optimization, we refer to the best use of computation and storage capacity at each of three different stages, namely, the mobile device, home server, and the central server. For example, the mobile device can play an important role in alerting the user in case of emergencies and therefore should be used for detecting the most urgent situations, especially in the absence of the wireless link. The home server typically has greater capacity and thus can perform much more complex tasks.

Another objective of this paper is to provide a secure and efficient scheme to meet the quality of service (QoS) requirements of medical and context data while they are transmitted from body sensors of mobile patients to health-care centers over body sensor networks and wireless networks of various types. In this regard, this paper also addresses two essential issues: (i) secure data transmission from body sensors to the mobile device over a body sensor network and (ii) efficient channel allocation and data security for transmission of medical and context-aware data to health-care center.

Earlier, we have proposed a wireless health monitoring system [22] that presents a three-tier architecture integrated to smart homes. Here, we enhance that architecture with (1) ZigBee profile descriptions for standards-based wireless communication. (2) A secure transmission mechanism that optimizes the use of system resources in medical monitoring applications. In addition, we provide the particulars of the system that we have developed that will be implemented in a pilot market. The next section presents a brief overview of the ZigBee technology. Then, we provide an introduction to ECG systems, which is the primary data we collect and process at the first stage of our development. The section that follows provides some specific aspects of our approach followed by some concluding remarks and discussion of our ongoing work.

## 2. MOBILE ECG SENSING, TRANSMISSION, AND ANALYSIS

**Figure 1:**
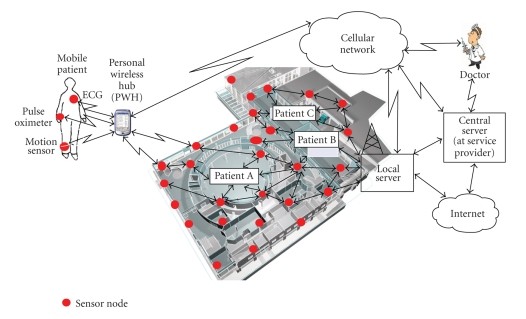
Wireless health monitoring in a smart environment.

Our system consists of several modules, each associated with a certain function as shown in Figure 1. Data collection module collects vital data, particularly ECG signal and provides local storage and transmission functionalities. The local server receives the data and preliminary analysis results and communicates with the central server as required. The central server follows the guidelines set by a particular service provider to initiate a sequence of response actions, including contacting the health professional.

Our system first measures the raw ECG signal from up to three electrodes and locally analyzes heart rate variability. If an arrhythmia risk is detected, an alert is transferred to the home server over the ZigBee network controller. At the same time, the raw ECG is measured and transmitted continuously to the home server, and then the home server analyzes the ECG records. If any anomaly is detected, patient's doctor is contacted. The ZigBee protocol does not have any transport layer functionality. Continuous transmission of the ECG data requires support for segmentation and reassembly, which is not offered by the current version of the ZigBee standard. We have implemented these functionalities at the application layer.

### 2.1. ECG basics

**Figure 2:**
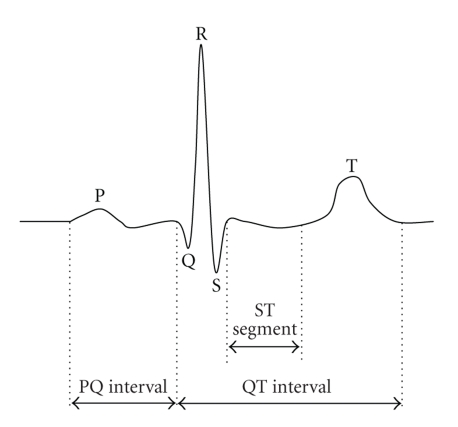
Illustration of the key ECG features.

The electrocardiogram is primarily a tool for evaluating the electrical events within the heart. The action potentials of cardiac muscle cells can be viewed as batteries that cause charge to move through the body fluids. These moving charges can be detected by recording electrodes at the skin surface. Figure 2 illustrates the typical lead II ECG where the active electrodes are placed on the right arm and left leg.

The first deflection, the P wave, corresponds to a current flow during atrial depolarization. Normal P waves have various shapes, from flat to sharply-peaked with amplitudes ranging from 0 to 0.3 mv. The PQ interval, extending from the beginning of the P wave to the first component of the QRS complex, corresponds to the depolarization of the atria, AV node, AV bundle and its branches, and the Purkinje system. The second deflection, the QRS complex, is due to ventricular depolarization. The final deflection, the T wave, is the result of ventricular repolarization. Atrial repolarization is usually not evident on the ECG, because it occurs at the same time as the QRS complex.

### 2.2. Sensing and analysis of ECG data

We have built a device for sensing and filtering ECG signals. Key steps consist of low-noise amplification, quantization, digital filters, and feature detection algorithms. The processed digital data is then sent to the home server over the ZigBee network.

**Figure 4:**
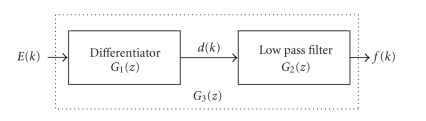
Filters for ECG signal conditioning.

Typical ECG signal level on the human body surface is around 2 mV. The AD converter used in our setup accepts voltages from 0 to 3 V. Therefore, we first add 1.5 V offset to center the ECG waveform prior to amplification. The amplified signal is then quantized to 8 bits by the ADC within the M16C microcontroller. The discrete waveform is passed through a differentiator and lowpass filter as shown in Figure 4, where *E*(*k*) represents the quantized ECG signal. The sampling rate in our implementation is 320 Hz. The filter transfer functions are as follows:

(1) $$G_{1}(z)=1-z^{-1}.$$

*G*_1_(*z*) is a differentiator filter; and is used to obtain slope of the QRS complex. *G*_2_(*z*) is a lowpass filter to avoid residual noise and intrinsic differentiation noise. Overall filter response maximizes the energy of the QRS complex and improves detection of R wave peaks.

Detection of QRS peaks has a great value in diagnosis of many medical anomalies. For detection of QRS peaks, numerous adaptive threshold detection techniques are used in the literature. Normalized amplitude distribution function of a smoothed ECG signal is used in [23] to detect whether a QRS complex is present within an observation interval. The slope of the distribution function becomes very sharp when the QRS complex does not exist inside the interval. Threshold value is set to be proportional to the R-R interval. In [24], two thresholds are set and the number of crossings are used to determine the QRS complex. The threshold values are determined adaptively from the signal amplitude. The higher threshold *γ_h_* is set to be *γ_H_* = max(0.5*P*_QRS_, gamma*_L_*), and the lower threshold *γ_L_* is set to be *γ_L_* = 0.75*γ_L_* + 0.25*P*_QRS_, where *P*_QRS_ indicates the peak of the latest QRS complex. These two algorithms are not very effective in presence of strong baseline drift.

In our work, a more robust adaptive threshold setting similar to that in [25] is used to detect QRS peaks. Note that due to severe baseline drifting and movement of patients, an ECG signal waveform may vary drastically from a heart beat to the next. With an adaptive threshold, probability of missing a QRS peak can be decreased.

In our platform, the first five seconds of the absolute value of the lowpass filtered digitized ECG data, *f*(*n*), is searched for its highest peak. Let us denote the magnitude of this peak as *p*[0]. Then, the threshold *τ* is initialized to *τ*[0] = *αp*[0], where *α* < 1. In our implementation, we set *α* = 0.65.

Let *p*[*i*] denote the first local peak of *f*(*n*) after a threshold crossing. After determining the slopes on both sides of *p*[*i*], the zero crossing between *p*[*i*] and the peak of the highest slope is chosen as the *i*th *R* wave peak location. The next threshold is set as follows:

(2) τ[i]=ατ[i−1]+(1−α)p[i−1].

If an R-to-R interval is measured as *β* times longer than the previous interval, where *β* > 1, a search is repeated only within that section of the ECG with a lower threshold to detect a possibly missed heart beat. We set *β* to 1.5 as a result of empirical observations.

The inverse of the interval between two consecutive *R* wave peaks gives the instantaneous heart rate. Their sequence shows how heart rate varies, which is another important medical data.

**Figure 5:**
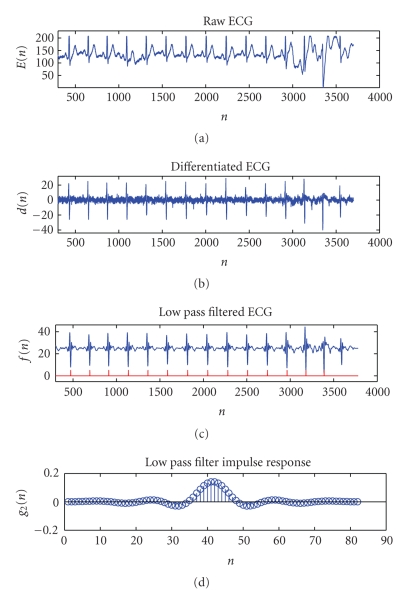
(a) Raw measured ECG signal, (b) ECG signal *d*(*n*) after the differentiator, (c) ECG signal *f*(*n*) after lowpass filtering. The detected R peaks are overlaid on the plot, and (d) impulse response of the lowpass filter, *g*_2_(*n*).

In Figure 5, a raw ECG trace and the output of the implemented R peak detector is shown. It is observed to be robust against baseline drifting caused by patient movements.

### 2.3. Secure transmission of vital sign data

Body sensors are used to sense vital sign data such as ECG for performing real-time health monitoring of mobile patients. Due to the transmission of sensitive medical data, it is imperative to build a strong security mechanism in order to protect the information transmitted as it may be susceptible to both active and passive attacks. Key distribution is central to any security mechanism based on cryptographic techniques. Whenever it is not possible to meet the power and computational requirements of asymmetric security techniques in small devices such as body sensors, symmetric key cryptography is employed to establish a secret session key for safeguarding data against various security attacks. Key management is fundamental to provide in body sensor networks (BSNs) because it provides and manages cryptographic keys to enable security services such as authentication, confidentiality, and integrity. Next, we will first introduce an attacker model and then present a secure key establishment and authentication algorithm for transmitting medical data from body sensors like ECG sensors to a handheld device of mobile patient, called personal wireless hub (PWH).

Our attacker model holds the following assumptions and properties. Prior to attaching body sensors to a mobile patient, we assume that they are not compromised and are embedded with a common global key, *K_CG_*, at a secure place like home. *K_CG_* is initially used to set up a session key at a secure place and then deleted. A compromised node of a BSN of a patient may eavesdrop on data being transmitted in its own BSN in order to break the session key. A compromised body sensor node of another close-by patient or intruder may eavesdrop on the medical data being transmitted in BSN of a neighboring patient. A compromised node from another BSN may try to inject false data into the BSN in order to force the PWH to establish a compromised session key. A compromised node may engage in replay attacks. A replay attack is a form of network attack in which a valid data transmission is maliciously or fraudulently repeated or delayed. This is carried out by a compromised node who intercepts the data and retransmits it at some later stage. This attack can be carried out by a compromised node which can be either internal or external to the BSN.

Our objective is now to establish two symmetric keys, namely *K*_PWH‐BSN_ and *K*_BSN‐BSN_ using an algorithm of three phases. The symmetric key *K*_PWH‐BSN_ is used to encrypt data between BSN sensors and PWH, while the symmetric key *K*_BSN‐BSN_ is employed among BSN sensors. The first phase is required to be implemented in a more secure place like home, though the second and third phases can be implemented at any place. In the *first phase*, PWH first generates an initialization key *K*_PWH_ using its random number generator, XORs the key *K*_PWH_ with the timing information of the last ECG signal sent by the BSN, and then sends the XORed result to the BSN sensors. At the end of the first phase, BSN sensors first recover the initialization key *K*_PWH_ using the same last ECG signal sent to PWH, and then agree on a nonce *N*_BSN_ among themselves. In the *second phase*, all BSN sensors compute the symmetric key *K*_PWH‐BSN_ = *K*_PWH_ ⊕ *N*_BSN_, encrypt *N*_BSN_ with *K*_PWH_, and send the encrypted message *E*_*K*_PWH__(*N*_BSN_) to PWH. At the end of the second phase, PWH first recovers *N*_BSN_ by decrypting *E*_*K*_PWH__(*N*_BSN_) with the initialization key *K*_PWH_ and then recovers the symmetric key *K*_PWH‐BSN_. In the *third phase*, PWH generates a nonce *N*_PWH_, computes the symmetric key *K*_BSN‐BSN_ = *K*_PWH_ ⊕ *N*_PWH_, encrypts *N*_PWH_ with *K*_PWH_, and sends the encrypted message *E*_*K*_PWH__(*N*_PWH_) to BSN sensors. At the end of the third phase, BSN sensors first recover *N*_PWH_ by decrypting *E*_*K*_PWH__(*N*_PWH_) with the initialization key *K*_PWH_ and then recover the symmetric key *K*_BSN‐BSN_. The established symmetric key can be used to encrypt data in those techniques supporting authentication, data integrity, and confidentiality. For instance, PWH can authenticate a BSN sensor node by comparing its aggregated timing information of heart beats with the heart beats' timing information sent by the BSN sensor node.

Once a session key is securely established between the body sensors and the PWH, the body sensors can use this session key for transmitting the data securely to the PWH. The established session key is known globally by all the body sensors and the PWH. Each body sensor also establishes a pairwise key with the PWH which is only known to the corresponding sensor and the PWH. To provide with added security, each body sensor provides with data confidentiality by encrypting the data with the session key and uses the pairwise key for data authentication. We can provide data confidentiality by encrypting the data with the session key established. To provide data integrity, a keyed-hash message authentication codes (HMACS) can be used along with a key as an input. To make it more secure from intruders, the key used for data confidentiality should be different from the key used to establish data integrity (to calculate the HMAC). The body sensors use the session key to encrypt the data to provide data confidentiality and use the pairwise key to calculate the HMAC to provide data integrity. Using this scheme, an intruder will need to have the knowledge of both the keys in order to spoof the PWH.

### 2.4. ZigBee for wireless sensing and transmission of medical data

Many medical applications will benefit from standards-based wireless technologies that are reliable, secure, and run on low power. Established standards for wireless applications, such as Bluetooth and IEEE 802.11, allow high transmission rates, but poses disadvantages such as high power consumption, application complexity, and cost. ZigBee networks on the other hand, are primarily intended for low duty cycle sensors, those active for less than 1% of the time for improved power consumption. For instance, an off-line node can connect to a network in about 30 milliseconds. Waking up a sleeping node takes about 15 milliseconds, as does accessing a channel and transmitting data. In addition, with their support of mesh networking and rapidly increasing popularity in wireless sensor network environments and smart homes make this networking technology a strong candidate for health applications as well.

**Figure 6:**
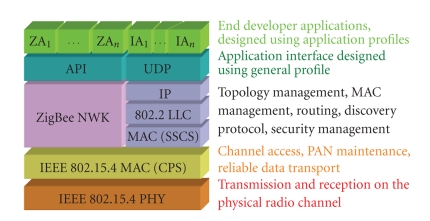
Illustration of ZigBee stack.

ZigBee is best described by referring to the 7-layer OSI model for layered communication systems Figure 6. The network name comes from the zigzagging path a bee (a data packet) follows to get from flower to flower (or node to node) [26]. The alliance specifies the bottom three layers (physical, data link, and network), as well as an Application Programming Interface (API) that allows end developers the ability to design custom applications that use the services provided by the lower layers. It should be noted that the ZigBee Alliance chose to use an existing data link and physical layer specifications. These are the recently published IEEE 802.15.4 standards for low-rate personal area networks. Complete descriptions of the protocols used in ZigBee can be found in [1, 27].

### 2.5. ZigBee network configuration: a medical profile proposal

Profiles in ZigBee networks provide a common protocol for communication within the network for a particular application to form an industry standard. So far, ZigBee Alliance has not issued an approved profile for use in health-care applications. In this section, we propose a proprietary profile description we have developed as part of this project that may also form a basis for future standardization efforts.

ZigBee network is configured in such a way that it uses one PAN per unit being monitored, that is, apartment, hospital section. Every device is configured as a ZigBee end-device. Several devices may coexist and report data simultaneously. In order to completely cover the monitored area, several additional ZigBee routers may be required. At the initial configuration, every router device discovers the route to the controller (PAN coordinator in our case). Every router then can respond to the route discovery request from the ECG device and does not need to rediscover the entire route.

The application software running on ZigBee devices is responsible for the creation of proper payload that carries corresponding commands, responses, and data. Once the payload is created, it is passed to the ZigBee APS layer for the transmission over the air using API provided by ZigBee stack manufacturer. Application endpoint has one incoming and two outgoing. Incoming cluster is used for command and control messages, one of the outgoing clusters is used to send response to control messages and the other to send raw ECG data. Node can receive command messages such as “start”, “stop” to control transmission, “setFQ” to set sample frequency, and others may be defined in the future. At 320 Hz, sample rate device produces 4 data packet per second. Depending on the hardware configuration (e.g., available RAM) some amount of data can be stored locally in case of temporary network failures.

ZigBee device endpoint consists of 2 incoming and 1 outgoing clusters. Outgoing cluster is for command and control interface. Incoming clusters receive command responses and ECG data. Upon receiving the data, ZigBee coordinator passes it to the server for further processing and analysis.

The application software running on ZigBee devices is responsible for creation of proper payload that carries corresponding commands, responses, and data. Once the payload is created, it is passed to the ZigBee APS layer for the transmission over the air using API provided by ZigBee stack manufacturer.

ZigBee Device Profile defines a set of commands and responses for a particular application. These are contained in *clusters* with the cluster identifiers enumerated for each command and response. Each ZigBee Device Profile message is then defined as a cluster. A cluster is a related collection of commands and attributes, which together define an interface to a specific functionality. Typically, the entity that stores the attributes of a cluster is referred to as the *server* of that cluster and an entity that affects or manipulates those attributes is referred to as the client of that cluster. Commands that allow devices to manipulate attributes, for example, the read or write attribute commands, are sent from a client device and received by the server device. Any response to those commands are sent from the server device and received by the client device. Conversely, the command that facilitates dynamic attribute reporting, that is, the report attribute command is sent from the server device (as typically this is where the attribute data itself is stored) and sent to the client device that has been bound to the server device. The clusters supported by an application object within an application profile are identified through a simple descriptor (see [1]), specified on each active endpoint of a device. In the simple descriptor, the application input cluster list contains the list of server clusters supported on the device, and the application output cluster list contains the list of client clusters supported on the device.

In our system, the server is the source node, that is, the mobile medical data collection device and client is the data collector node, that is, the home server. Our profile defines attributes and commands to configure ECG acquisition process. The server can receive the following commands: *SetSamplingFreq* to set sampling frequency of the analog signal at the ECG circuit, *Start* to start reporting ECG data, and *Stop* to stop reporting ECG data.

The server uses a reporting mechanism to send raw ECG data to the client for further processing. The format of the ECG data packet is summarized in Table 1. In the profile, Timestamp refers to the consecutive number that increments with every packet sent and allows to reconstruct ECG trace at the server in case packets come unordered or if some packets are missing; Freq is the sample frequency index, which is by default 320 Hz, and can be changed from the controller; ECG data is 80 bytes of digitized ECG signal, where each byte is a sample of the analog signal generated by the acquisition circuit.

## 3. EFFICIENT CHANNEL ALLOCATION FOR PATIENT DATA TRANSMISSION OVER WIRELESS NETWORKS

Future generation wireless networks will experience huge demands from mobile telemedicine applications. Mobile telemedicine will allow patients to do their daily activities while they are being monitored continuously anytime, anywhere. Typical telemedicine applications include transmission of ECG signals from the patient to the doctor, voice conversation between the doctor and the personnel in the emergency vehicle, transmission of X-rays, live video and medical images from the emergency vehicle, or the patient to the doctor at the health-care center. These applications require communication between a mobile patient and a health-care center or central server. But, these telemedicine applications will have to deal with the characteristics of wireless networks such as low bandwidth, channel fluctuations, and coverage changes. Further, a single network alone would not be able to meet the bandwidth requirements of applications at all locations. Fortunately, future mobile devices are expected to support multiple wireless interfaces, so that they can communicate through more than one wireless network at the same time. This allows the mobile applications to take advantage of various wireless networks, depending on their availability, channel conditions, coverage, and bandwidth. In this section, we present an efficient channel allocation algorithm to enable a PWH to allocate channels efficiently from wireless networks for mobile telemedicine applications.

Environmental factors can degrade the precision of ECG measurements. In addition, the human body can respond differently in cold temperatures and/or high altitudes, which may, for instance, prevent a finger pulse oximeter from taking an accurate measurement because blood flow to the fingers may get restricted. Therefore, not only ECG measurements, but also context information such as the time, location, environment and weather information associated with the ECG measurements should be sent from the mobile device to the server for analysis. Another important characteristic of ECG data is the difference in the periodicity and sporadic nature of the data. When the patient is under good conditions, the ECG data of the patient is typically sent to the health-care center periodically to monitor the condition of the patient. In addition to the periodic data, when the patient's condition deteriorates, sporadic emergency data needs to be sent to the health-care center, which may impact the periodicity and thus the wireless bandwidth requirement for the ECG data.

We assume that a patient exists in one of three states at a given moment with respect to a particular vital sign. At any given time, a patient's health status sign would be *GOOD*, *FAIR*, or *CRITICAL*. A patient's health status is treated as *GOOD* if the vital signs are stable and within normal limits. A patient's health status is treated as *FAIR* if the vital signs show slight instability and the patient may be uncomfortable. A patient whose vital sign data are unstable and not within normal limits, the patient's status is treated as *CRITICAL*. A simple way to predict a patient's status is through the use of thresholds. For example, we can measure the number of times that a vital sign value from a body sensor exceeds the acceptable value specified by the doctor. If out of *x* consecutive vital sign values, *y* of those vital sign values exceed the acceptable value, then the patient's status can be concluded as moving from *GOOD* to *FAIR* or *CRITICAL*. The exact way of determination of patient's status is specified by the medical doctor based on the patient's health history and the particular vital sign being measured. Determination of patient's status should also take into account the current activity level of the patient. That is, when the patient is exercising or doing some other highly intense activity, a different threshold should be used in determining the status of the patient from the threshold used under normal activity conditions. The current activity level of the patient can be determined from observing the outputs of body sensors that record the patient's speed, elevation, angle, room temperature, and so forth.

Wireless bandwidth can be reserved for periodic ECG data since we know the amount and time of occurrence of the data. In contrast to this, reserving bandwidth for emergency ECG data is not efficient in terms of resource usage. At the same time, emergency ECG data should be delivered on time without too much delay. To solve this problem, we propose a scheme, where the periodic ECG data is differentiated and if the differences in the periodic data exceeds a threshold, the PWH will start reserving bandwidth on the wireless networks in order to ensure the availability of the wireless bandwidth for any possible emergency ECG data. This reduces the wastage of bandwidth resources by not reserving bandwidth for emergency ECG data all the time. At the same time, by predicting the occurrence of an emergency situation and reserving resources beforehand based on the prediction, it also improves the probability of bandwidth availability under emergency situations.

We now present the dynamic channel allocation algorithm that is invoked whenever bandwidth is not available to satisfy an ECG data call request for emergency ECG data in a cellular network. The dynamic channel allocation algorithm presented here dynamically requests and migrates bandwidth from neighboring base stations. The base stations are responsible for bandwidth allocation. The load on all base stations is not going to be uniform. Also, every possible attempt should be made to not drop an ECG data call. Therefore, a dynamic and adaptive channel allocation scheme is essential, where channels are shared dynamically by various base stations of the same network access technology by adapting to the requirements of the clients.

The adaptive dynamic channel allocation (ADCA) algorithm presented here does the channel allocation to an ECG or a non-ECG call. A base station uses a different frequency, time slot, or code for each connection with a client. We also assume that each BS knows its neighboring BSs, that is, the network is already established and it remains fixed. The base stations do not move, however, the wireless clients can move from the coverage area of one base station to another. It is possible that some of base stations may become more loaded than the others. In such a situation, some channels have to be transferred from one base station to another.

The basic steps of the ADCA algorithm are as follows. On line 2, each base station computes and sends its call blocking probability for ECG and non-ECG calls to all of its neighbors. Based on the knowledge of its own call blocking probabilities and of that of its neighbors, on line 3, each base station determines whether a request can be made to borrow channels from any of the neighboring nodes. Based on the determination, neighboring channels are requested. If this base station receives a channel borrowing request from the neighboring node, it first checks if the number of free channels under the base station is greater than the threshold of free channels. If so, an appropriate free channel is moved from the base station to the requesting neighbor on line 5. On lines 6 to 9, a channel is allocated to an ECG call. An ECG call is assigned a channel as long as there are free channels available. On lines 10 to 13, a channel is allocated to a non-ECG call. A non-ECG call is allocated a channel only if the number of free channels is greater than the threshold of guard channels, TGC.

The ADCA algorithm makes use of two thresholds while assigning channels to an ECG data or a non-ECG data call. The objective is to ensure low call blocking probability for ECG data calls. Every base station maintains two thresholds, TFC and TGC, where TFC is the threshold of free channels and TGC is the threshold of guard channels for ECG data calls (TFC > TGC). Every base station periodically sends the call blocking probabilities of ECG and non-ECG calls to its neighbors. A non-ECG call is assigned a channel only when the number of free channels under the base station is greater than TGC, that is, TGC number of channels are always reserved for ECG data calls. In situations when an ECG data call cannot be allocated a channel even from the set of guard channels, the algorithm attempts to transfer a channel from a neighboring base station. A base station is allowed to transfer a channel only when the number of free channels under that base station is greater than TFC. Since TFC is greater than TGC, this transfer of channels does not affect the call blocking probability of ECG data calls under the base station that transfers the channels.

## 4. A SMART HOME SYSTEM WITH INTEGRATED MEDICAL MONITORING

In the previous sections, we have presented techniques for the capture, analysis, and secure transmission of ECG data for real-time monitoring of persons/patients in their homes. We envision that such techniques can work best in practice when they are integrated to existing platforms in homes such as wireless smart home systems. We have devised a three-stage approach where the vital signals are processed at the mobile device for immediate, life-threatening situations as described in the previous section. Here, we briefly present the processing at the next two levels: processing at the (local) home server and processing at the central service provider.

For improved performance, we perform the basic processing of ECG signals at the mobile device. This part entails the measurement pulse rate through QRS peak detection algorithm discussed earlier. This is done to facilitate a reliable warning in case of a network failure. Next, raw data basic analysis results are transmitted to the home server and central server as described in the next two sections.

### 4.1. Medical data processing at the home server 

Digitized ECG data is continuously transmitted to the home server via a ZigBee network. Additionally, results of the analysis at the mobile device are sent to the server and stored here for future reference. The goal is to provide a repository for more detailed analysis of the data by medical professionals or detection algorithms. In addition, the stored data is processed for more detailed and accurate analysis of ECG signals for detections such as Q-T interval and T wave detection.

The main responsibilities of the home server are: (1) coordinate the ZigBee in-home wireless network, (2) store incoming data, (3) conduct accurate and detailed analysis of the data, and (4) communicate with the central service provider for transmission of the data and notifications for detected anomalies. We are currently developing a Linux-based architecture housed on a PC-platform that allows remote access through a web-based interface. The server also is connected to the ZigBee network through a coordinator module connected via USB or serial port. Routers on the smart home network will be continuously powered and distributed throughout the home, possibly one for each room. An existing ZigBee network normally used for lighting or security can be used for this purpose as well. The data repository is made available for future use by service provider as well as the user and is backed up against losses through a data warehousing service.

### 4.2. Medical data processing at the central server

Continuous recording and analysis of ECG data provides an excellent basis for automated detection as well as professional diagnosis of many cardiological symptoms. According to our model, the last and the third piece is the central data processing center where servers as well as medical personnel can provide a variety of services such as storage, early diagnosis, and in-home care. The home server transmits periodic reports and makes stored data available to the central server.

A key point for the central server processing is the optimization of the use of resources at the home server resources and the central server. As the number of users increase, the central server can allocate only a limited amount of computational capacity to each user. Therefore, data analysis is performed at the home server as much as possible.

The central server also functions as an entry point for the professional staff to monitor the data and reports generated by the home server. In addition, the alerts initiated by the mobile device are transmitted to the central server through the home server. The central server also keeps records of all transactions through an event management system.

## 5. CONCLUSION

We have presented a real-time and secure architecture for health monitoring in smart homes using ZigBee technology. Our research has outlined many specific issues that relate to a collection of emerging technologies, namely, wireless communication, secure transmission, and processing of medical data within the context of “smart environments”. In particular, we believe that our work has made the following contributions:

- description of a ZigBee profile for transmission of ECG data over a wireless sensor network,
- a security model for secure transmission of data over wireless sensor network,
- an efficient mechanism for channel allocation in wireless networks to transmit medical data, and
- a three-tier architecture for optimized analysis of data using an adaptive mechanism that maximizes the utility of processing and computational capacity at each of three stages.

Our project continues towards completion of the implementation of local and central server components for an end-to-end service. In addition, we are expanding the collection of information to other modalities such as oxygen, temperature, and glucose level measurements.

## Figures and Tables

**Figure 3:**
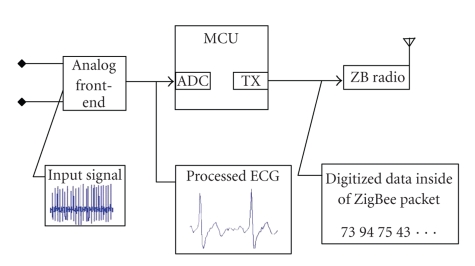
Block diagram of the ECG measurement platform.

**Algorithm 1:**
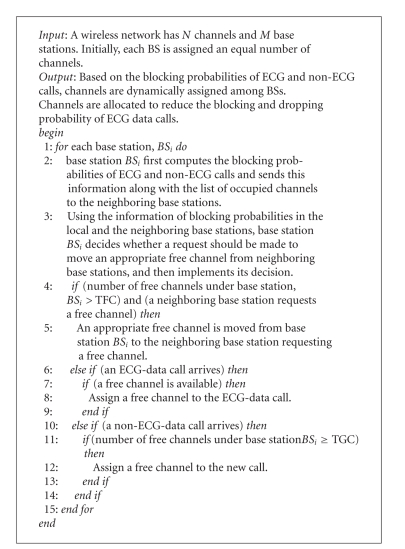
Algorithm ADCA.

**Table 1:** ZigBee ECG profile.

Type	ZigBee headers	Timestamp	Freq.	ECG Data
Length	Variable	4 bytes	1 byte	80 bytes
Example		00 00 00 00	00	00 00 00 ⋯ 00
